# Sensing and feeling: an overview

**DOI:** 10.1098/rstb.2023.0242

**Published:** 2024-08-26

**Authors:** Giulia L. Poerio, Hirohito M. Kondo, Brian C. J. Moore

**Affiliations:** ^1^ School of Psychology, University of Sussex, Falmer BN1 9QH, UK; ^2^ School of Psychology, Chukyo University, Nagoya, Aichi 466-8666, Japan; ^3^ Cambridge Hearing Group, Department of Psychology, University of Cambridge, Downing Street, Cambridge CB2 3EB, UK

**Keywords:** emotion, vision, hearing, touch, interoception

## Abstract

Emotional experiences are driven, in part, by the way we process and integrate information from different sensory modalities. Understanding how perceptual and emotional systems interact to give rise to subjective feelings is an important, complex and challenging issue, requiring new approaches and integrative thinking that fuses the fundamentals of low-level sensory perception with higher-level cognitive and affective processes. The Theme Issue ‘Sensing and feeling: an integrative approach to sensory processing and emotional experience’ showcases fifteen theoretical, empirical, and review articles from experts working at the intersection of perception and emotion, encompassing multiple sensory systems (visual, auditory, tactile and interoceptive), clinical and non-clinical perspectives (e.g. affective disorders and hearing loss), contextual and social perspectives, and complex emotional experiences in special populations. Articles in Part 1 emphasize recent advances across fields in sensory and emotion science and give insights into future directions. Each article in Part 2 provides more detailed and specific methodological approaches or theoretical models, and focuses on basic mechanisms linking sensation to emotional experience.

This article is part of the theme issue ‘Sensing and feeling: an integrative approach to sensory processing and emotional experience’.

## 1. Introduction

Feelings are central to human experience, shaping essential aspects of how our lives are lived, including our judgements, decisions, thoughts and social interactions. Although this is functionally adaptive, difficulties in perception, reactivity and regulation of emotions are core diagnostic features of mental health disorders and consequently are often the target of intervention [1]. Understanding how feelings emerge and when they are disrupted can provide important insights into the development, progression and treatment of mental disorders. Such understanding also can shed important light on the many functional aspects of feelings and emotional experience, e.g. for facilitating social interaction, well-being, pleasure and feelings of contentment.

Recent advances in affective neuroscience argue for a view of emotion as a constructive process; that is, emotions emerge as a meaning-making mechanism integrating incoming sensory inputs from our external senses and the body, i.e. exteroception and interoception [2–4]. While this view emphasizes the important role of sensory processing in the generation of emotions, very little research in the field of sensory systems explicitly examines the interaction between sensory perception and emotion [5], the link between sensory processing and emotion being implicitly assumed. This Theme Issue features contributions that *explicitly* shed light on the connection between sensory processing systems and emotional experience. Our aim is to provide an illustrative and diverse collection of articles to highlight important ways in which research on sensation, perception and emotion would benefit from closer integration and cross-fertilisation, in contrast to the ‘siloed’ approach that has dominated thus far. This Theme Issue brings together contributions from leading experts that cover a range of important topics in this emerging multi-disciplinary field, encompassing:

(1) Different sensory modalities, not just the visual and auditory modalities that tend to dominate sensory systems research, but also touch, taste/smell, proprioception (sensing the body's position in space) and interoception (sensing the internal state of the body);
(2) clinical perspectives, examining the role of sensation and emotion in affective and sensory-specific disorders, including potential developmental origins and the role of social context, and;
(3) non-clinical perspectives that highlight the importance of sensory processing in the experience of more complex positive emotions, such as aesthetic appreciation and music.

The contributions in this Theme Issue consist of review and opinion articles as well as primary research from a broad range of perspectives and methods. This includes areas such as experimental psychology, cognitive neuroscience, clinical science and theories of aesthetics. We have organized the collection in two parts. Part 1 is intended to serve as a general introduction to core issues addressed in later empirical articles in the collection, consisting of three theoretical pieces that highlight the roles of: (1) interoception (the body), (2) sensory features of external stimuli, and (3) individual differences in emotional experience. Part 2 features articles that expand on these core issues, mostly with empirical research papers showcasing recent research and advances at the interface between sensory experience and emotion.

## 2. Part 1—theoretical perspectives on sensing and feeling

Damasio and Damasio [6] take up the challenge of defining and explaining consciousness in terms of sensing and feeling. They argue that interoception—sensing the internal state of the body—is the basis for consciousness and subjective experience. This capacity distinguishes organisms with neural systems capable of interoceptive processesing from those solely capable of sensing their external environment without a nervous system capable of representing interoceptive stimuli. Consciousness, in their view, is attributable to homeostatic feelings (e.g. hunger, thirst, desire, breathing and cardiac function) that result from complex interactions between neural (mind) and non-neural (bodily) events. In this way, consciousness results from the automatic and spontaneous experience of feelings, which provide continuous information about the internal state of an organism and internal (subjective) representations of the external world. It is this combined information, derived from the body and external world, that provides information about what the organism needs and allows for adaptive and deliberate behaviour and survival.

Maurer and Maurer [7] similarly consider sensing and feeling from an evolutionary standpoint, but with a focus on external sensory stimulation and aesthetic responses. In their opinion piece, they propose that the ability to efficiently detect changes in sensory input is both essential for species survival and the mechanism that explains aesthetic pleasure and displeasure. They argue that ‘aesthetic preferences are neither sublime nor even uniquely human’ and instead are an incidental consequence of a common biological and neurophysiological mechanism that allows organisms to detect subtle deviations from what is ‘normal’ or average. Reviewing evidence from a range of sensory realms (taste, smell and vision), across different species, and considering aesthetic categories such as music and dance, the authors describe how neural systems adapt to detect deviations from normality from birth and how these form the basis of aesthetic responses. Central to Maurer and Maurer's argument is the idea that features of sensory stimuli are vital for understanding subsequent emotional responses, with deviations from statistical regularities of a stimulus attracting attention and resulting in positive or negative emotional responses (depending on an individual's context or previous experiences).

While the first two introductory articles focus on universal neurophysiological mechanisms bridging sensing and feeling, Lionetti and Pluess [8] emphasize the need to consider individual differences and special populations for understanding connections between sensing and feeling, a key theme explored further in later papers of this collection. The authors offer a new theoretically informed and empirically grounded framework to explain how highly sensitive individuals, who make up around a third of the population, differ in their experience and processing of emotion. Lionetti and Pluess review literature across three areas, exploring how sensitivity affects emotion processing, emotional reactivity and emotion regulation, shaped by both genetic and environmental factors. Heightened sensitivity is linked to the ability to perceive subtleties in the environment, higher emotional reactivity (both positive and negative) appearing to explain why some people show stronger emotional reactions than others, with important implications for well-being.

## 3. Part 2—recent advances in sensing and feeling

### (a) The body and mind

Expanding on the central theme of the role of interoception and the body in emotional experience, Poerio *et al*. [9] explore the role of attention to the body in the everyday experiences of emotion. They report the results of an experience-sampling study in which participants were prompted at random intervals by text message ten times daily over five or seven days. On each occasion, participants reported on their ‘in the moment experiences’ including interoceptive attention to 21 bodily sensations (e.g. heartbeat, breathing, hunger, thirst and pain), their emotional experiences (fatigue, negative valence and tension), and, as a control, their attention to auditory sensations, classified as exteroceptive. Interoceptive signals were reported as the object of attention about 20% of the time. Paying greater attention to interoceptive, but not to exteroceptive, signals was associated with more negative valence and greater fatigue. Overall, from an ecological view, these results support the idea that interoceptive attention is important for the generation of emotional experiences in everyday life. The results suggest that emotional experience is influenced by the extent of the attention allocated to internal signals.

Eccles *et al*. [10] similarly explore the fundamental role of the body in emotional experience, but do so with reference to proprioception as well as interoception. As the authors note, links between proprioception and emotional experience are under-researched. In their paper, they describe and provide evidence for a conceptual model in which poorly predicted proprioceptive signals (proprioceptive ‘surprise’) enhance the generation of interoceptive prediction errors, which then generate emotional experiences. This model was tested in the context of neurodivergence (atypical populations), hypermobility (which predisposes individuals to proprioceptive imprecision) and emotional dysregulation. These data support the model, suggesting that regulation of the body's positions and actions may influence feelings and sensations through proprioceptive and interoceptive mechanisms. The findings underscore the importance of understanding interactions between the brain and body for much-needed interventional innovation in mental health, especially those that target proprioceptive imprecision and move beyond a ‘one-size-fits-all’ approach to treatment.

Focusing on a single, relatively underexplored interoceptive modality—breathing—Bischoff *et al*. [11] explore the role of cortical and subcortical brain regions involved in voluntary respiration and its link to anxiety. Twenty patients with lesions to the frontal, insular, temporal cortex and/or basal ganglia and the same number of matched healthy controls were compared for their performance of separate breathing and motor tasks, in which they had to time their breathing (inhalation/exhalation) or motor movements (rotating a dial) based on a visual cue. Patients with brain lesions reported heighted anxiety across both tasks, especially during irregular or rapid breathing. They also had a reduced ability to voluntarily control both breathing and movement. One implication of these results is that the neural circuits involved in complex motor movement may also be necessary for optimizing voluntary breathing control. Overall, the study helps to clarify the role of the frontal, temporal or limbic regions in abnormal voluntary respiratory and motor regulation and tachypnea (abnormally rapid breathing) related anxiety, highlighting the role of the forebrain in affective and motor responses during breathing.

The relationship between emotional and bodily processes is bi-directional, as elegantly shown by Gillmeister *et al*. [12], who explore how being exposed to others' emotional states affects somatosensory resonance—the shared neural representation of states such as pain between self and others. Crucially, they investigated whether neural processes differed for individuals with an altered sense of bodily self, i.e. feelings of disembodiment, which are found in the psychological condition of depersonalisation-derealisation (DP-DR). The authors conducted electroencephalography (EEG) to probe how DP-DR levels modulated perceptual mechanisms underlying emotional face recognition and mirror touch (experiencing tactile sensations when seeing someone else being touched). Emotional processing in those with DP-DR was weak, but somatosensory resonance increased with others’ bodily sensations following exposure to the negative emotions of others. This led to the intriguing suggestion that experiencing oneself as less psychologically distinct from others makes people with DP-DR more susceptible to the effects of others' negative emotions on subsequent sensory processing. Overall, the findings suggest that feeling disconnected from one's body and self might protect from the threat of negative feelings, which may be worsened in DP-DR through self–other confusion and contribute to symptoms of emotional numbness in the disorder.

### (b) Influence of sensory stimuli on emotion across vision, taste, touch and hearing

Taschereau-Dumouchel *et al*. [13] test two competing ideas to clarify the role of early visual perception in the subjective experience of fear. They address the question: Are there evolutionary hardwired threat-detection mechanisms within early perception that lead to the subjective experience of fear, or do fearful stimuli tend to have common visual properties that do not, in themselves, represent the subjective experience of fear? To address this question, functional magnetic resonance imaging (fMRI) responses to visual stimuli were compared between people who reported high or low fear levels in response to the stimuli. Participants viewed 3600 pictures of animals and objects. The results showed that participant ‘fear profiles’ could be accurately decoded from activity in ventral visual areas, regardless of whether participants reported the stimuli as fearful. Fearful stimuli could also be decoded by artificial neural networks that were not trained to identify threat or fear but rather just to identify objects. Taken together, the results suggest that early visual representations do not actually encode fear, but instead reflect statistically common visual properties that tend to be perceived as threatening. The authors also produced synthetic images that represented common visual properties of fear-evoking stimuli (e.g. bees, worms and spiders). Although visual responses to such synthetic stimuli did not differ depending on subjective fear, synchronisation between visual regions and prefrontal areas did; participants who reported being fearful of certain stimuli showed enhanced synchronisation between visual and prefrontal pathways. The authors argue that fear experiences may depend on relevant visual information triggering mechanisms in prefrontal regions, rather than in limbic regions, which in turn drives the subjective experience of fear (or appraisal of stimuli as fearful).

Prescott and Spinelli [14] address a different sensory modality—taste—and the features of food that affect emotional responding. Acceptance or rejection of food by both children and adults is related to physiological and psychological arousal, high arousal being associated with greater rejection. The authors review the characteristics of food that can lead to high arousal (and therefore rejection). These characteristics include intensity (strength of smell or flavour), novelty, complexity and perceived dangerousness. The paper goes further to show that arousal and the propensity to become aroused strongly influence responses to stimuli generally, but especially foods, influencing food preference and choices. Some individuals have ‘food neophobia (FN)’—a fear of new foods. FN is common in young children, for whom it may confer a degree of protection by promoting avoidance of potentially toxic items that might be mistaken for food. For some individuals, FN persists into adulthood and appears to be associated with the experience of unpleasantly high arousal in response to novel foods. Physiological changes, such as increased pulse and respiration rates and sweating, occur for individuals with FN when simply looking at pictures of foods. The authors suggest that the effect of arousal on food choices is one aspect of a general sensory sensitivity.

Sensory modalities not only enable the experience of emotion, they also underscore how emotions are socially communicated, a point nicely illustrated by Maallo *et al*. [15]. They investigated the neural basis of emotional communication through touch. Close friends or romantic partners took part in an fMRI study examining whether different patterns of touch can be decoded into a variety of emotions by the receiver of the touch, on both behavioural and neural levels. For each partnership, the ‘sender’ communicated emotional messages (e.g. happiness, love and sadness) through touch to the forearm, and the ‘receiver’ tried to determine the message they thought their partner intended to communicate. Participants were able to decode emotional meaning from their partner's touch well above chance levels. Using multivariate decoder algorithms, the authors were able to discriminate the intent and interpretation of different emotional touch categories based only on fMRI activity across primary somatosensory cortex. A broader network of non-sensory regions, including parietal, visual, opercular and premotor cortex, predicted the performance of receivers of touch, suggesting a role beyond peripheral input in determining how emotion meaning is understood from interpersonal touch.

A different approach to understanding how sensory features affect emotional experience was taken by Moore [16]. He considered the emotional consequences of a reduced or no ability to detect auditory features in music. Music can evoke a wide range of emotions, such as happiness, sadness, tension, relief and excitement. Several studies have been devoted to investigating what acoustic features of musical sounds are associated with the perception of emotion. However, people with hearing loss and people with cochlear implants (CIs) have an impaired ability to discriminate some of the features that convey emotion in music. Moore reviews how changes in perceptual abilities affect the perception of emotion in music. Although people with acquired mild to moderate hearing loss have nearly normal perception of emotions in music, those with severe loss experience difficulties. This is especially true when hearing loss is congenital, perhaps due to limited ability to learn the acoustical features in music that convey emotion. Hearing aids can alleviate the adverse effects of hearing loss on emotional music perception, but not entirely and they seem to work best for simpler melodies compared to complex pieces. CI users can discriminate rhythms but have difficulties in judging pitch and discriminating melodic contours, which may result partly from altered perception of dynamic changes in sound level. An open question is whether features of hearing aids and CIs intended to improve audibility do so at the expense of listeners being able to extract emotional meaning from complex sounds such as music.

### (c) Special populations

Articles in the final section of the issue focus on two sensory-emotional special populations in which there is burgeoning scientific interest: those experiencing Autonomous Sensory Meridian Responses (ASMR), and those with misophonia. ASMR is a non-universal sensory-emotional experience felt as pleasurable tingling sensations originating in the crown of the head and a sense of relaxation. Misophonia is characterised by strong negative emotional reactions to certain sounds, usually produced by the actions of people or animals, such as sounds produced by chewing or sniffing. The negative reactions occur even when the sounds are not especially loud. These two special populations provide unique opportunities to explore interactions between low-level perceptual processes and top-down cognitive processes in the generation of both positive and negative emotional experiences.

Although sensory stimuli that most commonly elicit ASMR are tactile (e.g. playing with hair and stroking), many people commonly consume ASMR content with audio-visual triggers to alleviate stress and induce relaxation. Terashima *et al*. [17] describe the acoustic features of sounds in video recordings that tend to produce ASMR tingling sensations, studying particularly the role of amplitude fluctuations in different frequency regions, which determine the sound ‘texture’. Participants viewed video recordings with sounds that were known to be effective in eliciting the ASMR and also viewed ‘nature’ videos. They were instructed to continuously rate their perceptual experience, either of tingling sensations or of pleasant feelings. The results showed that tingling and pleasantness were somewhat independent. For example, ‘nature’ videos tended to produce pleasant feelings but did not lead to much tingling. The sensation of tingling was best predicted by a lower amplitude around the 5-kHz frequency range over a time period 1500 to 750 ms before the response was made. The results may be useful for designing sound textures that enhance the likelihood of evoking the ASMR, with possible practical applications in increasing well-being.

Lin and Kondo [18] provide an overview of what is currently known about the neural basis of ASMR by reviewing neuroimaging studies. They concude that common brain regions may underlie ASMR experiences. The authors go further by identifying the common and distinct neural circuits associated with sensory-emotional phenomena similar to ASMR, encompassing synaesthesia, sensory processing sensitivity and misophonia. Two key regions—the insula and anterior cingulate cortex—emerge as common regions involved across the reviewed sensory-emotional phenomena. The authors conclude by emphasizing the role that these regions may play in social cognition and emotion regulation processes.

In a similar vein, Mednicoff *et al*. [19] explore commonalities between sensory-emotional phenomena—focusing on how people with misophonia respond to ASMR-evoking and musical stimuli. In their online study, the authors assessed the emotional reactivity of misophonic adults to a range of audio-visual emotion-inducing stimuli. Participants watched videos meant to induce either misophonia, ASMR or musical chills, and were asked to click a button whenever they had an emotional reaction to a video and to rate the emotional valence (strength of emotion) and arousal of each video. Reactions to misophonia-inducing videos were correlated with reactions to the ASMR- and chill-inducing videos, perhaps indicating individual differences in the overall strength of both positive and negative emotional responses. The results could reflect a general phenotype of stronger emotional reactivity to meaningful sensory inputs.

In the last article in this section, Berger *et al*. [20] nicely situate emotional responses to sensory stimuli in a broader social context. They argue that for understanding of misophonia, it is necessary to consider not just the characteristics of ‘trigger’ sounds, but also the context in which they are perceived, the emotions that they evoke, and the strategies that are used to cope with trigger sounds. They point out that the extent to which a potential trigger sound elicits a negative reaction depends on the perceived source of the sound, even when the source is incorrectly identified. They propose that misophonia should be understood within a framework of social perception and cognition. Consistent with this, when misophonia is triggered, this leads to the activation of brain areas that are thought to be involved in social processing. The authors discuss potential therapeutic implications of their social interpretation of misophonia.

## 4. Concluding remarks

This Theme Issue presents the cross-disciplinary development of research and theory on the sensation–emotion interface from a broad range of perspecitives and methods. Articles in this issue cover areas such as experimental psychology, cognitive neuroscience and clinical science, using a wide range of techniques: psychophysics, surveys, intensive longditudinal methods, functional neuroimaging (fMRI and EEG) and computational modelling. This broad approach is important for an integrated understanding of findings from different disciplines to explain the intimate connection between our sensory and emotional systems. Taken together, the issue sheds new light on how our brain, body and external worlds interact to give rise to emotional experience, with important implications for advancing our knowledge of the neurobiological development and maintenance of emotional and sensory-specific disorders and, ultimately, for evidence-based interventions. We hope that the special issue galvanises further theoretical and conceptual integration and convergence between researchers and practitioners interested in sensory perception, emotion and well-being.

## Data Availability

This article has no additional data.
